# Association of *NCF2*, *IKZF1*, *IRF8*, *IFIH1*, and *TYK2* with Systemic Lupus Erythematosus

**DOI:** 10.1371/journal.pgen.1002341

**Published:** 2011-10-27

**Authors:** Deborah S. Cunninghame Graham, David L. Morris, Tushar R. Bhangale, Lindsey A. Criswell, Ann-Christine Syvänen, Lars Rönnblom, Timothy W. Behrens, Robert R. Graham, Timothy J. Vyse

**Affiliations:** 1 Department of Medical and Molecular Genetics, Division of Genetics and Molecular Medicine, School of Medicine, King's College London, London, United Kingdom; 2 Academic Department of Rheumatology, Division of Immunology, Infection, and Inflammatory Diseases, School of Medicine, King's College London, London, United Kingdom; 3 Department of Bioinformatics and Computational Biology, Genentech, South San Francisco, California, United States of America; 4 Rosalind Russell Medical Research Center for Arthritis, Division of Rheumatology, University of California San Francisco, San Francisco, California, United States of America; 5 Molecular Medicine, Department of Medical Sciences, Uppsala University, Uppsala, Sweden; 6 Section of Rheumatology, Department of Medical Sciences, Uppsala University, Uppsala, Sweden; 7 ITGR Human Genetics Group, Genentech, San Francisco, California, United States of America; University of Oxford, United Kingdom

## Abstract

Systemic lupus erythematosus (SLE) is a complex trait characterised by the production of a range of auto-antibodies and a diverse set of clinical phenotypes. Currently, ∼8% of the genetic contribution to SLE in Europeans is known, following publication of several moderate-sized genome-wide (GW) association studies, which identified loci with a strong effect (OR>1.3). In order to identify additional genes contributing to SLE susceptibility, we conducted a replication study in a UK dataset (870 cases, 5,551 controls) of 23 variants that showed moderate-risk for lupus in previous studies. Association analysis in the UK dataset and subsequent meta-analysis with the published data identified five SLE susceptibility genes reaching genome-wide levels of significance (*P_comb_*<5×10^−8^): *NCF2* (*P*_comb_ = 2.87×10^−11^), *IKZF1* (*P*_comb_ = 2.33×10^−9^), *IRF8* (*P*_comb_ = 1.24×10^−8^), *IFIH1* (*P*_comb_ = 1.63×10^−8^), and *TYK2* (*P*_comb_ = 3.88×10^−8^). Each of the five new loci identified here can be mapped into interferon signalling pathways, which are known to play a key role in the pathogenesis of SLE. These results increase the number of established susceptibility genes for lupus to ∼30 and validate the importance of using large datasets to confirm associations of loci which moderately increase the risk for disease.

## Author Summary

Genome-wide association studies have revolutionised our ability to identify common susceptibility alleles for systemic lupus erythematosus (SLE). In complex diseases such as SLE, where many different genes make a modest contribution to disease susceptibility, it is necessary to perform large-scale association studies to combine results from several datasets, to have sufficient power to identify highly significant novel loci (*P*<5×10^−8^). Using a large SLE collection of 870 UK SLE cases and 5,551 UK unaffected individuals, we firstly replicated ten moderate-risk alleles (*P*<0.05) from a US–Swedish study of 3,273 SLE cases and 12,188 healthy controls. Combining our results with the US-Swedish data identified five new loci, which crossed the level for genome-wide significance: *NCF2* (neutrophil cytosolic factor 2), *IKZF1* (Ikaros family zinc-finger 1), *IRF8* (interferon regulatory factor 8), *IFIH1* (interferon-induced helicase C domain-containing protein 1), and *TYK2* (tyrosine kinase 2). Each of these five genes regulates a different aspect of the immune response and contributes to the production of type-I and type-II interferons. Although further studies will be required to identify the causal alleles within these loci, the confirmation of five new susceptibility genes for lupus makes a significant step forward in our understanding of the genetic contribution to SLE.

## Introduction

Systemic lupus erythematosus (SLE) is a relapsing-remitting complex trait which most commonly affects women of child-bearing age, with a ratio of 9∶1 in female to males. The disease prevalence varies with ethnicity, being more prevalent in non-European populations (approximately 1∶500 in populations with African ancestry and 1∶2500 in Northern Europeans) [1]. The condition is characterised by the production of a diverse range of auto-antibodies against serological, intra-cellular, nucleic acid and cell surface antigens [2]. The wide-ranging clinical phenotypes include skin rash, neuropsychiatric and musculosketal symptoms and lupus nephritis, which may be partially mediated by the extensive deposition of immune complexes. Today, thanks to improved treatments, the 10-year survival rate after diagnosis has increased to 90%, with lower survival rates being related to disease severity or complications from treatment [3]. Increased understanding of the underlying genetic basis for lupus is of key importance in improving the prognosis for lupus patients.

Until recently, the genetic basis of lupus remained largely undetermined, with only about ∼8% of the genetic contribution known [4]. However, within the last three years, tremendous progress has been made in defining novel loci, through three moderate-sized genome-wide association studies in European American cohorts and a replication study in a US-Swedish cohort [5]–[7]. The loci previously identified for SLE include genes involved in the innate immune response (eg. *IRF5*), T and B cell signalling (eg. *STAT4*, *TNFSF4* and *BLK*), autophagy/apoptosis (eg. *ATG5*), ubiquitinylation (*UBE2L3*, *TNAIP3*, *TNIP1*) and phagocytosis (*ITGAM*, *FCGR3A* and *FCGR3B*). All of these pathways are of potential importance in lupus pathogenesis [8]–[10].

To date, a total of 1729 independent SLE cases have been subjected to genome-wide association genotyping using three genotyping platforms: Illumina 317 K BeadChip [5], Illumina 550 K BeadChip [6] and Affymetrix 500 K array [7]. There is currently no published meta-analysis of these datasets.

The aim of the current work was to perform a replication study using our UK SLE cohort on loci that showed some evidence for association in previous studies in order to extend the list of confirmed susceptibility genes for lupus.

## Results

To identify additional susceptibility loci for SLE, we first identified the independent genetic variants that showed moderate risk (*5×10^−3^<P*>5×10^−8^) in a combined US-Swedish dataset comprising 3273 SLE cases and 12188 controls [4]. We then genotyped 27 independent SNPs in a replication cohort of 905 UK SLE cases and 5551 UK control samples (Table 1), that included both British 1958 Birth Cohort samples and additional controls from the WTCCC2 project.

**Table 1:** SLE Case-Control Study Cohorts used in the study.

Study	Origin of Samples	SLE cases			Control samples			
		UK^a^	US^c^	SWE^d^	UK (B58BCC)^a^	UK (WTCCC2)^b^	US^c^	SWE^d^
**UK**	UK	870			68	5483		
**US GWAS**	Gateva *et al* (2009) [4]		1310				7859	
**US replication**	Gateva *et al* (2009) [4]		1129				2991	
**SWE replication**	Gateva *et al* (2009) [4]			834				1338
**Total**		870	2439	834	5551		10850	1338

Each of the seven groups of samples included in this manuscript was independent from each other. In the UK population, direct genotyping was carried out on 905 UK cases**^a^** and samples from the British Birth Control Cohort^a^ (B58BCC). A total of 905 UK SLE cases were typed and for the analysis, 35 cases were removed following QC. Genotypes from the WTCCC2**^b^** were used as out-of-study controls. The published data used for the meta-analysis described in this current manuscript was derived from US and Swedish samples. The US cohort**^c^** consisted of samples included in a GWAS and additional non-GWAS'd samples used just for the replication study, as described by Gateva *et al*. (2009) [4]. Full details of the Swedish (SWE)**^b^** replication samples are also described in Gateva *et al.* (2009) [4].

For the 27 genotyped SNPs, 10 variants which had not been genotyped by the WTCCC2 project, were imputed using IMPUTE2 [11]. This imputation was performed using CEPH HapMap samples as the phased reference sequence and the boundary of the surrounding haplotype blocks used to demarcate the imputation interval. The subsequent association analysis excluded two of these ten imputed SNPs because they had less than 95% certainty for the imputation (Table S2). In the US/SWE dataset, imputation of selected SNPs not genotyped previously [4] was performed using IMPUTE1 for HapMap. Phase II CEU sample haplotypes were used as reference with subsequent association analysis performed using SNPTEST and a genomic control factor (lambda-GC) values of: 1.05 (US dataset) and 1.10 (SWE dataset) after correction for population stratification.

In the UK replication sample by performing allelic association analysis using PLINK for the 23 SNPs passing QC (Tables S2 and S3), we demonstrated moderate association (*P*≤0.05) for twelve variants - with a lambda-GC of 1.01 following ancestry correction (see Table 2 and Table 3). Under the null hypothesis, only 1 of the 23 loci would be expected to have P≤0.05. The observed enrichment of associated SLE genes in the UK dataset suggested that many of these loci were likely to be true-positive associations.

**Table 2:** Novel SNPs showing genome-wide significance (*P* = 5×10^−8^) in SLE following meta-analysis of UK, US, and Swedish cohorts.

MARKER	Locus	Risk allele	UK population870 *cases, 5551 controls*			*P* value (US/SWE)^a^ *3273 cases, 12188 controls*		Combined AnalysisFisher's test*P* value
			Freq risk allele^b^	OR	*P* value	OR	*P* value	
rs10911363	*NCF2*	*T*	*0.27*	1.23	3.02×10^−4^	1.19	9.50×10^−8^	2.87×10^−11^
rs2366293	*IKZF1*	*G*	*0.14*	1.20	8.77×10^−3^	1.23	2.66×10^−7^ c	2.33×10^−9^
rs2280381	*IRF8*	*A*	*0.62*	1.11	0.0491	1.16	2.53×10^−7^ c	1.24×10^−8^
rs1990760	*IFIH1*	*T*	*0.61*	1.11	0.0487	1.17	3.34×10^−7^	1.63×10^−8^
rs280519	*TYK2*	*A*	*0.47*	1.20	5.24×10^−4^	1.16	7.40×10^−5^ d	3.88×10^−8^

a For sample numbers see reference [4] and Table S1 (US GWAS: 1310 cases and 7859 controls; US replication cohort: 1129 cases and 2991 controls; Swedish replication cohort: 834 cases and 1388 controls).

b The risk allele frequency was calculated in control individuals.

c Unpublished data.

d The combined P value was calculated from imputed genotypes in the US GWAS dataset and direct genotyping in the US and SWE replication datasets.

**Table 3:** Additional SNPs showing association with SLE in the UK, US, and Swedish cohorts.

MARKER	Locus	Risk allele	UK population*870 cases, 5551 controls*			*P* value (US/SWE)^a^ *3273 cases, 12188 controls*		Combined AnalysisFisher's test*P* value
			Freq risk allele^b^	OR	*P* value	OR	*P* value	
***SNPs showing borderline genome-wide significance (5×10^−8^<P<1×10^−7^)***								
rs428073	*TAOK3*	*T*	*0.68*	1.10	0.0900	1.17	7.70×10^−7^	6.93×10^−8^
rs17696736	*C12ORF30*	*G*	*0.43*	1.21	2.05×10^−4^	1.08	4.00×10^−4^	8.20×10^−8^
rs9782955	*LYST*	*C*	*0.75*	1.05	0.450	1.18	4.60×10^−7^	2.07×10^−7^
rs1874791	*IL12RB2*	*T*	*0.19*	1.03	0.619	1.21	3.40×10^−7^	2.10×10^−7^
rs497273	*UNQ1887*	*G*	*0.64*	1.02	0.698	1.16	8.20×10^−7^	5.72×10^−7^
***SNPs showing evidence for association in combined dataset (P>1×10^−7^)***								
rs1861525	*CYCS*	*G*	*0.05*	1.08	0.555	1.27	1.90×10^−6^	1.05×10^−6^
rs11951576	*POLS*	*C*	*0.69*	1.01	0.907	1.14	4.60×10^−6^	4.17×10^−6^
rs641153	*CFB*	*C*	*0.92*	1.24	0.0356	1.30	1.40×10^−4^	4.98×10^−6^
rs6438700	*CASR*	*C*	*0.82*	1.01	0.947	1.18	5.50×10^−6^	5.20×10^−6^
rs3212227	*IL12B*	*A*	*0.81*	1.15	0.0369	1.13	1.70×10^−4^	6.27×10^−6^
rs3184504	*SH2B3*	*T*	*0.49*	1.14	0.0156	1.11	5.57×10^−4^	8.69×10^−6^
rs12708716	*CLEC16A*	*A*	*0.65*	1.10	0.0996	1.16	1.60×10^−4^	1.59×10^−5^
rs10516487	*BANK1*	*C*	*0.68*	1.12	0.0500	1.11	8.30×10^−4^	4.15×10^−5^
rs10156091	*ICA1*	*T*	*0.11*	1.04	0.604	1.16	6.50×10^−4^	3.93×10^−4^
rs2022013	*NMNAT2*	*A*	*0.58*	1.05	0.326	1.09	0.0015	4.89×10^−4^

a For sample numbers see reference [4] and Table S1 (US GWAS: 1310 cases and 7859 controls; US replication cohort: 1129 cases and 2991 controls; Swedish replication cohort: 834 cases and 1388 controls).

b The risk allele frequency was calculated in control individuals.

We confirmed the similarity of odds-ratios (Het *P* value) and direction of the effect between the UK and US-SWE datasets (Table S4) and then performed a meta-analysis using Fisher's combined *P*-value (see Materials and Methods). This meta-analysis revealed five novel associated loci with *P*<5×10^−8^ (Table 2): *NCF2* (neutrophil cytosolic factor 2) (rs10911363, *P*_comb_ = 2.87×10^−11^, OR_comb_ = 1.19); *IKZF1* (Ikaros family zinc-finger 1) (rs2366293, *P*_comb_ = 2.33×10^−9^, OR_comb_ = 1.24); *IRF8* (interferon regulatory factor 8) (rs2280381, *P*_comb_ = 1.24×10^−8^, OR_comb_ = 1.16); *IFIH1* (interferon-induced helicase C domain-containing protein 1) (rs1990760, *P*_comb_ = 1.63×10^−8^, OR_comb_ = 1.15) and *TYK2* (tyrosine kinase 2) (rs280519, *P*_comb_ = 3.88×10^−8^, OR_comb_ = 1.17)(Table 1). The strength of these associations was similar to those found from a weighted meta-analysis, using the METAL programme (Table S4). A case-only analysis using PLINK in the combined UK/US/SWE dataset revealed no non-additive interactions between the five newly associated variants (*P*>0.05). These new SLE loci are discussed in more detail below and with additional information in Text S1.

Three of the SNPs tested were for loci that had shown genome-wide levels of significance in other SLE GWAS studies (Table S5). In the UK cohort we found further support for the association at *JAZF1* (rs849142 *P*_UK_ = 0.0243, OR_UK_ = 1.13) and identified a third associated variant in the first intron of *TNIP1* (rs6889239 *P*_UK_ = 9.06×10^−6^, OR_UK_ = 1.30), which is in strong LD (r^2^ = 0.895) with both the previous report in Europeans [4] and in perfect LD with a third SNP (rs10036748), first reported in a Chinese GWAS [12]. All three variants in *TNIP1* are located within a 661 bp region of intron 1. We did not replicate the previous association with *IL10* (rs3024505, *P*_UK_ = 0.209 OR_UK_ = 1.09) (Table S5).

These analyses increased the evidence of association for a number of additional loci that had shown borderline significance in the original US/SWE GWAS (Table 3), including *CFB*, *C12ORF30*, *SH2B3*, and *IL12B*. Genotyping of additional samples will be required to determine if the association signals shown in Table 3 represent confirmed genetic loci for SLE.

## Discussion

The work presented here confirms five new susceptibility loci for SLE at the level of genome-wide significance (*P*<5×10^−8^). Each of the associated variants lie within, or close to, the coding sequence for genes with known roles in immune regulation: *NCF2*, *IKZF1*, *IRF8*, *IFIH1* and *TYK2*. Interestingly, each of these genes has been implicated in interferon signalling. While the interferons have classically been defined as anti-viral cytokines, recent studies have suggested an important role for interferon in the pathophysiology of SLE [13]. While most evidence points to the role of type I interferon in SLE [14] there is substantial data suggesting that type II interferon (IFNγ) is also involved in SLE pathogenesis [15].

*NCF2* (neutrophil cytosolic factor 2) (1q25), is induced by IFNγ and specifically expressed in a number of immune-cell types, including B-cells. Our data suggest that the *NCF2* association is independent from the previously reported signal in the neighbouring locus *NMNAT2*, [5] because we found no evidence of strong LD between the genotyped SNP within *NMNAT2* (rs2022013) and that in *NCF2* (rs10911363) (r^2^ = 0.136). Logistic regression in the UK replication cohort confirmed that *NMNAT2* did not contribute to the association at *NCF2* (*P* = 0.777).

**Figure 1:**
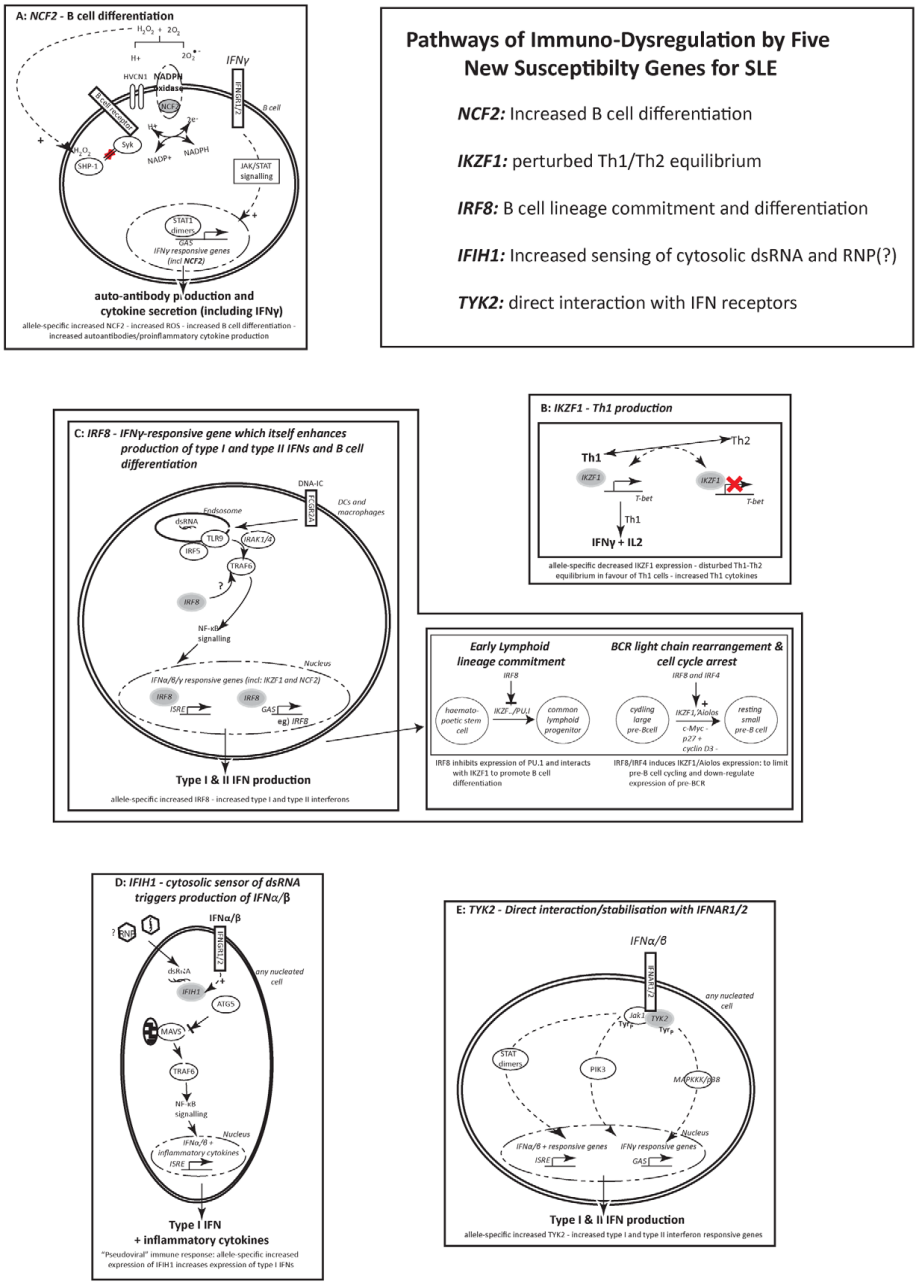
Pathways of immuno-dysregulation by five new susceptibility genes for SLE. The figure shows the interferon-related pathways involving *NCF2*, *IKZF1*, *IRF8*, *IFIH1* and *TYK2* and and the ways in which these pathways may contribute to lupus susceptibility. In SLE these five genes contribute to increasing the levels of type-I and –II interferons, imbalances in Th1/Th2 related to disease severity, perturbations in B cell physiology and production of a diverse set of auto-antibodies.

*NCF2*, as a cytosolic subunit of NADPH-oxidase, may have a role in the increased production of the free radicals characterising B-cell activation [16] (Figure 1) which increases auto-antibody levels and may suggest a mechanism for the involvement of *NCF2* as a susceptibility gene for SLE.

**Figure 2:**
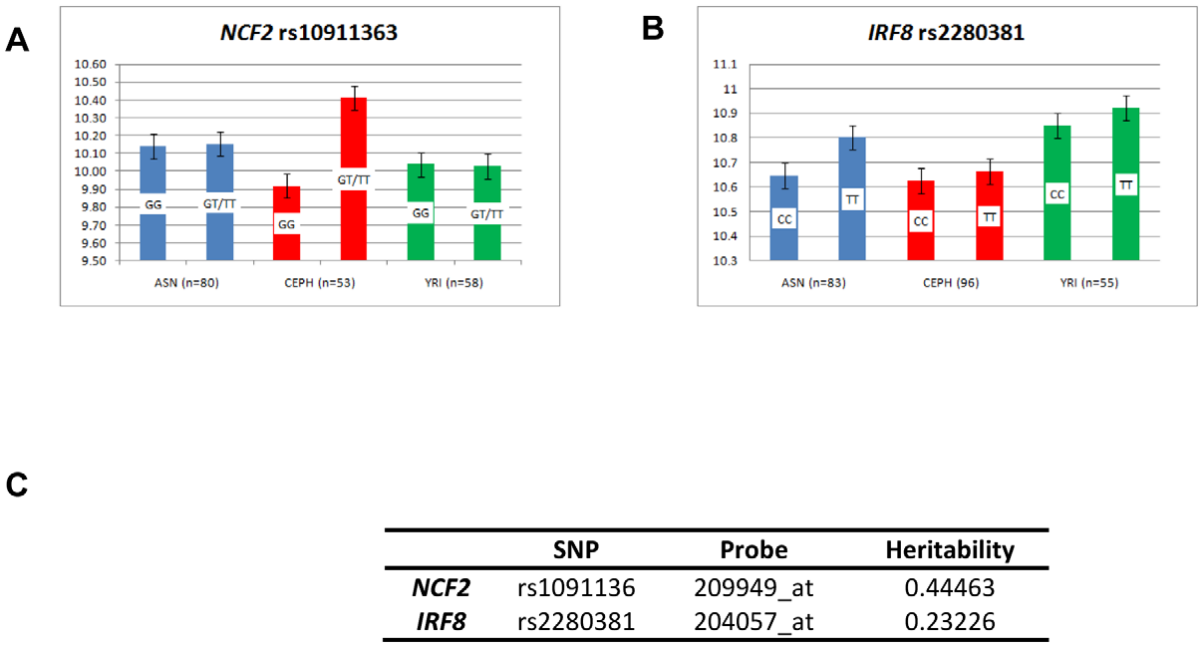
Expression pattern in EBV-transformed lymphoblastoid cell lines. Regression analysis, as described in the materials and methods, was performed on publically available genotype data from EBV-transformed B cells which were part of the HAPMAP collection and expression data on the same individuals taken from the GEO database. Four populations were used: CEPH, YRI and CHB/JPT (ASN) [36]. The GEO dataset was GSE12526 and the expression probes were: A) *NCF2* (209949_at), B) *IRF8* (204057_at). For each graph, the mean expression per risk (R) allele and that for the non-risk (r) allele was plotted for each population. The alleles are listed on each bar and for each SNP, the total number of individuals for which there was both genotype and expression data are quoted for the three populations analysed. C) Heritability estimates for each locus were taken from the mRNA by SNP browser (http://www.sph.umich.edu/csg/liang/asthma/).

There are allele-specific significant expression differences for rs10911363, following a recessive model of basal expression for the risk T allele of rs10911363 in CEPH individuals but not in YRI and ASN (CHB+JPT) HapMap cohorts (*P_CEPH_ = *0.03) (Figure 2A). There is also a significant difference in gene expression for a variant (rs3845466) located 2 kb away from rs10911363 in intron 2 of *NCF2* (Figure S2A), using lymphoblastoid cell lines (LCLs) from umbilical cords of 75 individuals which were taken from the GENEVAR collection (*P* = 0.0228). The population-specific nature of this correlation could be because of local differences in the pattern of LD within *NCF2* between the CEU, YRI and ASN (CHB+JPT) HapMap cohorts. These population specific differences in LD may be between the genotyped SNP and an unknown causal allele(s) responsible for an expression difference seen in multiple ethnic backgrounds or between the genotyped marker and an unknown causal allele(s) exhibiting population-specific differences in gene expression itself. However, it will be necessary to confirm these findings in primary cells and tissues, because the EBV-transformed B cells model system may not entirely reflect the physiological conditions in peripheral B cells. Indeed a recent report showed that there may be systematic changes in gene expression within EBV-transformed B cells [17]. Nevertheless, with this caveat in mind, and taking each locus on a case-by-case basis, the model-based approach can provide important insights into measurement of transcript levels in *ex vivo* cells. For example, the increases in transcript levels that we initially observed in EBV-LCLs for *OX40L*, were also confirmed in peripheral blood B cells [18].

*IKZF1* (Ikaros family zinc-finger 1) (17p14.3) is a transcription factor essential for dendritic cell and lymphocyte development. The association with rs2366293 is supported by a report of a second associated variant, rs921916 (*P*_comb_ = 2.0×10^−6^) [4], found 860 bp away from rs2362293, which is in strong LD with rs2366293 (r^2^ = −0.746, D′ = 0.925) (Figure S2B). A third SNP, rs4917014, located ∼200 kb upstream of *IKZF1*, showed association with SLE in a Chinese GWAS (*P_GWAS_* = 2.93×10^−06^), but it was a separate signal from the European SNPs (r^2^<0.0002) [9], [12]. IKZF1 has a role in the production of IFNγ, by blocking the production of the Th1 master-regulator T-bet (Figure 1). The shifted Th1/Th2 equilibrium (in favour of Th1 cells) increases the levels of IFNγ directly [19] rather than indirectly as a result of cross-talk between the type-I and type-II IFN signalling pathways eg) via type-I interferon mediated activation of STAT1 homodimers, which are the primary means of signalling from IFNγ [20] and have recently been shown to be associated with SLE in a Swedish cohort [21].

The transcription factor *IRF8* (interferon regulatory factor 8) (16q24.1), shows immune-cell restricted expression. rs2280381 is found 64 kb downstream of *IRF8*, and is in LD with the coding region (Figure S2C), but independent from a susceptibility allele for multiple sclerosis (rs17445836), 1 kb away [22]. The lupus variant influences *IRF8* gene expression, since LCLs from three HapMap cohorts, showed a significant increase in *IRF8* transcript levels in homozygotes for the risk allele (TT) compared to homozygotes for the non-risk allele (CC) (*P* = 0.045) (Figure 2A). IRF8 also has a key role in regulating the differentiation of myeloid and B-cells and in mice, IRF8 restricts myeloid cell differentiation but promotes B-cell differentiation [23](Figure 1).

*IFIH1* (interferon-induced helicase C domain-containing protein 1) (2q24.3) is an ubiquitiously expressed, cytoplasmic sensor of dsRNA. The SLE risk allele for rs1990760 (Table 1) is identical to that previously reported in two organ-specific autoimmune diseases: T1D [24] and Graves' Disease [25]. Regression analysis using publically available genotype data from HapMap and expression data from GEO dataset GSE12526 revealed that individuals who were homozygous for the common risk T allele of rs1990760 had significantly higher *IFIH1* transcript levels compared to individuals who were homozygous for the non-risk allele (*P* = 0.8.19×10^−5^) (Figure S3B). Furthermore, a recent paper showed that the presence of the risk T allele of rs1990760 was correlated with increased levels of IFN-induced gene expression, in lupus patients who were positive for anti-dsDNA antibodies [26]. Another report demonstrated that *IFIHI* was rapidly up-regulated by type-I IFNs (Figure 1), and that IFIH1 signalled downstream through NF-κB, to further increase IFN-α production [27].

*TYK2* (tyrosine kinase 2) (19p13.2) phosphorylates the receptor subunits of cytokine receptors, including type-I IFN receptors which are found on all nucleated cells, leading to increased production of type I interferon responsive genes (Figure 1). The significant association in intron 11 TYK2 for rs280519 in our UK cohort (*P* = 5.24×10^−4^) crossed the threshold for genome-wide significance when combined with the US/Swedish cohort. The association for rs280519 increases the genetic evidence for the involvement of TYK2 reported in a smaller UK family-based SLE cohort [28]. There was an earlier report, using a Swedish/Finnish population, of association in *TYK2*. This Swedish/Finnish study showed association for a missense mutation in exon 8 (rs2304256) (*P*_comb_ = 5.60×10^−5^, *P*_Swe_ = 9.60×10^−5^) [29]. The Swedish individuals used in the earlier analysis are a subset of the Swedish individuals analysed for this current manuscript and rs2304526 is in moderate LD with the *TYK2* SNP that we typed in this current study - rs280519 (r^2^_CEPH-HapMap_ = 0.373). The association for rs2304256 was replicated in a second moderate sized European study [30], but not in the GWAS from the SLEGEN consortium [5]. In preliminary analysis in UK cases and controls, there are data to support the fact that rs280519 is enriched in SLE cases (n = 345) with renal disease compared to healthy controls (n = 5551) (P = 0.033).

There were variants in several loci for which we have found evidence of association (*P*<0.05) in our UK cohort, but which did not reach genome-wide significance in the combined analysis. One of these variants was rs17696736, located in intron 15 of *C12ORF30* (*MDM20*). This protein is a subunit of N-acetyltransferase complex B (NatB), and may promote apoptosis by reducing cell cycle progression [31]. In the joint cohort, rs17696736 was in LD (r^2^ = 0.625) with a second variant on chromosome 12q24, a missense W262R allele (rs3184504) in the lymphocyte adaptor protein *SH2B3*. SH2B3 facilitates T-cell activation by mediating the interaction between the T-cell receptor and T cell signalling molecules [32]. Both MDM20 and SH2B3 are also associated with T1D [33], and *SH2B3* is additionally associated with celiac disease [34] and both myocardial infarction and asthma [35]. The associated variant within *IL12B*, rs3212227, is located in the 3′ UTR region, and the SLE risk allele is the same as previously reported for psoriasis [36]. *IL12B* encodes for the larger subunit (p40) of two cytokines, IL12 and IL23, and thereby contributes to both Th1 [37] and Th17 [38] immune responses.

In summary, we have identified five new genes contributing to SLE risk: *NCF2*, *IKZF1*, *IRF8*, *IFIH1* and *TYK2*. Dense fine-mapping and/or genomic re-sequencing of each locus will be required to reveal the functional alleles for each gene with respect to immune dysregulation in lupus. Taken together, these findings further support an important role of interferon pathway dysregulation in lupus pathogenesis.

## Materials and Methods

### Ethics statement

The ethical approval for the study was obtained from the London Multi-Centre Research Ethics Committee (London MREC).

### Details of the UK SLE samples in the study cohort

All of the 905 UK SLE cases conformed to the ACR criteria for SLE [39] with a diagnosis of SLE being established by telephone interview, health questionnaire and details from clinical notes. Written consent was obtained from all participants. Genomic DNA from the UK samples was isolated from anti-coagulated whole blood by a standard phenol-chloroform extraction.

### Genotyping methodology

Each of the 27 SNPs were genotyped on a custom Illumina chip, using the BeadXpress platform at the Oklahoma Medical Research Foundation (OMRF), Oklahoma. The panel of ancestry informative markers was typed independently on an Illumina platform at Gen-Probe, Livingstone.

### Power calculations

Power calculations were performed in the UK case-control dataset for each of the markers tested, using the algorithm described by Purcell *et al* [40]. Taking into account varying minor allele frequencies for the risk alleles and the differences in effect size (OR), and by employing a population prevalence of 0.002 and D′ of 1, with an type I error rates, alpha = 0.05, each of the SNPs showing novel genome-wide significance in the meta-analysis showed a power of >48% (2) to detect an association in our cohort.

### Quality control of genotyping

Markers were excluded from the analysis if they showed a genotyping success rate of less than 95% or had a Hardy-Weinberg P value in the B58BCC control samples of less than *P* = 0.001. A total of 21 cases were removed from the final analysis due to low percentage genotyping (<95%). All samples were filtered for cryptic relatedness and duplication using an identity by state test in PLINK (PI_HAT score >0.1). The full list of genotyped variants and the results of the QC analysis are shown in (Table S3).

### Correction for ancestry

A total of 35887 markers, distributed across each autosome, were selected for ancestry correction in the UK case-control cohort, these markers had all been typed as part of the HapMap project and on the WTCCC2 samples. The 35887 SNPs were chosen from a set of Illumina 317 K markers pruned for LD (r^2^<0.25) after removing regions of known extended LD, including the extended MHC and the region covering the inverted repeat on chromosome 8 (*pers commun*. David Morris, King's College and Kim Taylor, UCSF). This list of AIMs is available directly from the corresponding author, Professor Timothy Vyse.

The EIGENSTRAT PCA analysis was performed on the UK cases and also the control samples, both from the genotyped B58BCC and the WTCCC2 out-of-study controls. The eleven populations from HapMap3 were used as external references. Each SNP included in the PCA analysis showed >95% genotyping in the each dataset. Following EIGENSTRAT analysis, a graph was plotted of PC1 against PC2 for all the cases and controls in the UK study cohort (Figure S1). Individuals were only retained for association analysis if the values for their first two principal components fell within 6 SD of the mean for the CEPH HapMap samples. The genomic inflation factor (lambda-GC) for each population was calculated using PLINK.

### Statistical analysis

All sample genotype and phenotype data was managed by, and analysis files generated with BC/SNPmax and BC/CLIN software (Biocomputing Platforms Ltd, Finland).

The imputation intervals for each imputed variant, defined as the bounds of the haplotype blocks, calculated using the Gabriel algorithm in Haploview, (for details of the intervals see Table S2). For SNPs which were not genotyped as part of the WTCCC2 project, we performed imputation using a method described by Marchini *et al* [11] to generate the missing genotypes for case-control association analysis. Each un-typed variant from our list of tested SNPs, was imputed in the WTCCC2 samples, using HAPMAP as the phased reference sequence. The LD pattern around each un-typed variant was examined using the CEPH cohort from HapMap. The boundaries of the haplotype blocks were determined using the default settings for the Gabriel et al algorithm in Haploview. For each imputed variant, these haplotype boundaries were used to define the boundaries of the imputation interval (Table S2). Only SNPs with greater than a 95% certainty in imputation, assessed using the quality score from the IMPUTE2 output file, were used for subsequent analysis.

Allelic association testing, using UK SLE cases with either genotyped control samples or imputed genotypes, was carried out using PLINK (http://pngu.mgh.harvard.edu/~purcell/plink/).

Prior to performing the meta-analysis, the heterogeneity of odds ratios was tested using METAL and the Cochran-Mantel-Haenszel test (Table S4). SNPs with *P* value<0.001 between the two studies were discarded. Combined analysis of *P* values generated in the UK samples with those from the US/SWE cohort in published data [4] was conducted using Fisher's combined *P* value and with a meta-analysis using the programme METAL, which weighted the effect size, based on the inverse of the standard error.

To determine whether there was any allele-specific effect on the level of gene expression, we used publically available genotype data on unrelated EBV-transformed B cells (CEU, YRI and CHB/JPT individuals which were part of the HapMap project) and expression data from the same individuals (GSE12526, GEO database) [41]. For each locus, which reached genome-wide significance by meta-analysis, we categorised the expression data based on the SNP genotype for the respective associated variant (homozygote risk allele, heterozygote and homozygous non-risk allele). The significance of the correlation between genotype and expression level was then calculated using logistic regression analysis in SNPTEST, using gender as a covariate.

Interactions between the five SNPs reaching genome-wide significance following meta-analysis, were assessed using the epistatic option in PLINK. To maximize the power of this test, we restricted our analysis to the SLE affected individuals from the combined US/SWE/UK cohort.

## Supporting Information

Figure S1 Correction for population substructure. A total of 16 UK cases and 8 out-of study controls were removed from the final analysis because their PC1 and PC2 values were greater than 6SD away from the mean of the PC1 and PC2 values for the CEPH external reference individuals (−0.00197>value>0.012807). The 870 SLE cases and 5551 control individuals retained for association analysis are located within the ellipse on the graph.
(EPS)

Figure S2 Patterns of LD around the five SLE susceptibility genes reaching genome-wide levels of significance. Patterns of linkage disequilibrium in CEU individuals taken from HapMap, using positions from data release 27 phase II+III Feb09, NCBI assembly dbSNP 126: (A) 200 kb around rs10911363 (Chr 1:181,716,380-181,916,379); (B) 155.8 kb region around rs2366293 (Chr 7:50,120,474-50,276,274); (C) 100 kb region around the gap between IRF8 and rs2280381 (Chr 16:84500150-845600149); (D) 250 kb region around rs1990760 (Chr 2:162,700,000-162,949,999); (E) 200 kb region around rs280519 (Chr 19: 10,233,933-10433933).
(PDF)

Figure S3 Variants showing a trend for changes in expression in EBV-transformed lymphoblastoid cell lines. Regression analysis, as described in the materials and methods, was performed on publically available genotype data from EBV-transformed B cells which were part of the HAPMAP collection and expression data on the same individuals taken from the GEO database. Four populations were used: CEPH, YRI and CHB/JPT (ASN) [2]. The GEO dataset was GSE12526 and the expression probes were: A) *IKZF1* (205039_s_at), B) *IFIH1* (219209_at), C) *TYK2* (205546_s_at). For each graph, the mean expression per risk (R) allele and that for the non-risk (r) allele was plotted for each population. The alleles are listed on each bar and for each SNP, the total number of individuals for which there was both genotype and expression data are quoted for the three populations analysed. D) Heritability estimates for each locus were taken from the mRNA by SNP browser (http://www.sph.umich.edu/csg/liang/asthma/).
(EPS)

Table S1 Composition of the study cohorts used. Each of the seven groups of samples included in this manuscript was independent from each other. In the UK population, direct genotyping was carried out on UK cases^a^ and samples from the British Birth Control Cohort^a^ (B58BCC). Genotypes from the WTCCC2^b^ were used as out-of-study controls. The published data used n the meta-analysis described in this current manuscript was derived from US and Swedish samples. The US cohort^c^ consisted of samples included in a GWAS and additional non-GWAS'd samples used just for the replication study, as described by Gateva *et al*. (2009) [4]. Full details of the Swedish (SWE)^b^ replication samples are also described in Gateva et al (2009) [4].
(DOC)

Table S2 Quality control of genotype data and imputation boundaries for WTCCC2 control samples. The position of each variant (column “Pos”) is given using NCBI Build 36. The number of WTCCC2 control samples is given for each variant in the column marked “WTCCC2 samples.”
(DOC)

Table S3 Power calculations. ^a^Novel associations in this study (5×10^−8^). The OR, as a measure of effect size was taken from the case-control association study. The power was calculated according to Purcell et al 2003 (http://bioinformatics.oxfordjournals.org/content/19/1/149.full.pdfhtml), using a disease prevalence of 0.0002. The risk allele frequency was calculated in both cases and controls. GRR (AB) = (AB_case_/AA_case)_/(AB_control_/AA_control_) and GRR (AA) = (BB_case_/AA_case_)/(BB_control_/AA_control_).
(DOC)

Table S4 Results of weighted meta-analysis using METAL and calculation of combined OR. The total number of individuals included in the meta-analysis was: 870 UK SLE cases and 5,551 UK control samples and 3,273 SLE cases and 12,188 controls taken from the US/SWE out-of-study cohort [1]. The risk allele frequency quoted is that from the UK cases. The column marked Het P-value represents the test for heterogeneity of odds ratios between the UK and published dataset and the column marked OR_comb_ represents the OR in the combined dataset, calculated using METAL. The column marked Direction of Effect demonstrates that the effect for each quoted allele is the same for the UK and US/SWE datasets.
(DOC)

Table S5 Association Analysis in UK, and US-Swedish Populations for Markers Previously Showing Genome-Wide Significance (*P*<5×10^−8^). ^a^ For sample numbers see reference [1] and Table S1 (US GWAS: 1,310 cases and 7,859 controls; US replication cohort: 1,129 cases and 2,991 controls; Swedish replication cohort: 834 cases and 1,388 controls). ^b^ The risk allele frequency was calculated in control individuals. ^c^ Unpublished data.
(DOC)

Text S1 This file contains supplementary genomic and functional details about the five SLE susceptibility genes reaching genome-wide levels of significance.
(DOC)
