# Supplementary material for: Association of NCF2, IKZF1, IRF8, IFIH1, and TYK2 with Systemic Lupus Erythematosus
Source: PLoS Genet. 2011 Oct 27;7(10):e1002341. doi: 10.1371/journal.pgen.1002341 (PMC3203198; doi:10.1371/journal.pgen.1002341)
Supplement: Figure S2 — Patterns of LD around the five SLE susceptibility genes reaching genome-wide levels of significance. Patterns of linkage disequilibrium in CEU individuals taken from HapMap, using positions from data release 27 phase II+III Feb09, NCBI assembly dbSNP 126: (A) 200 kb around rs10911363 (Chr 1:181,716,380-181,916,379); (B) 155.8 kb region around rs2366293 (Chr 7:50,120,474-50,276,274); (C) 100 kb region around the gap between IRF8 and rs2280381 (Chr 16:84500150-845600149); (D) 250 kb region around rs1990760 (Chr 2:162,700,000-162,949,999); (E) 200 kb region around rs280519 (Chr 19: 10,233,933-10433933). (PDF) [file pgen.1002341.s002.pdf]

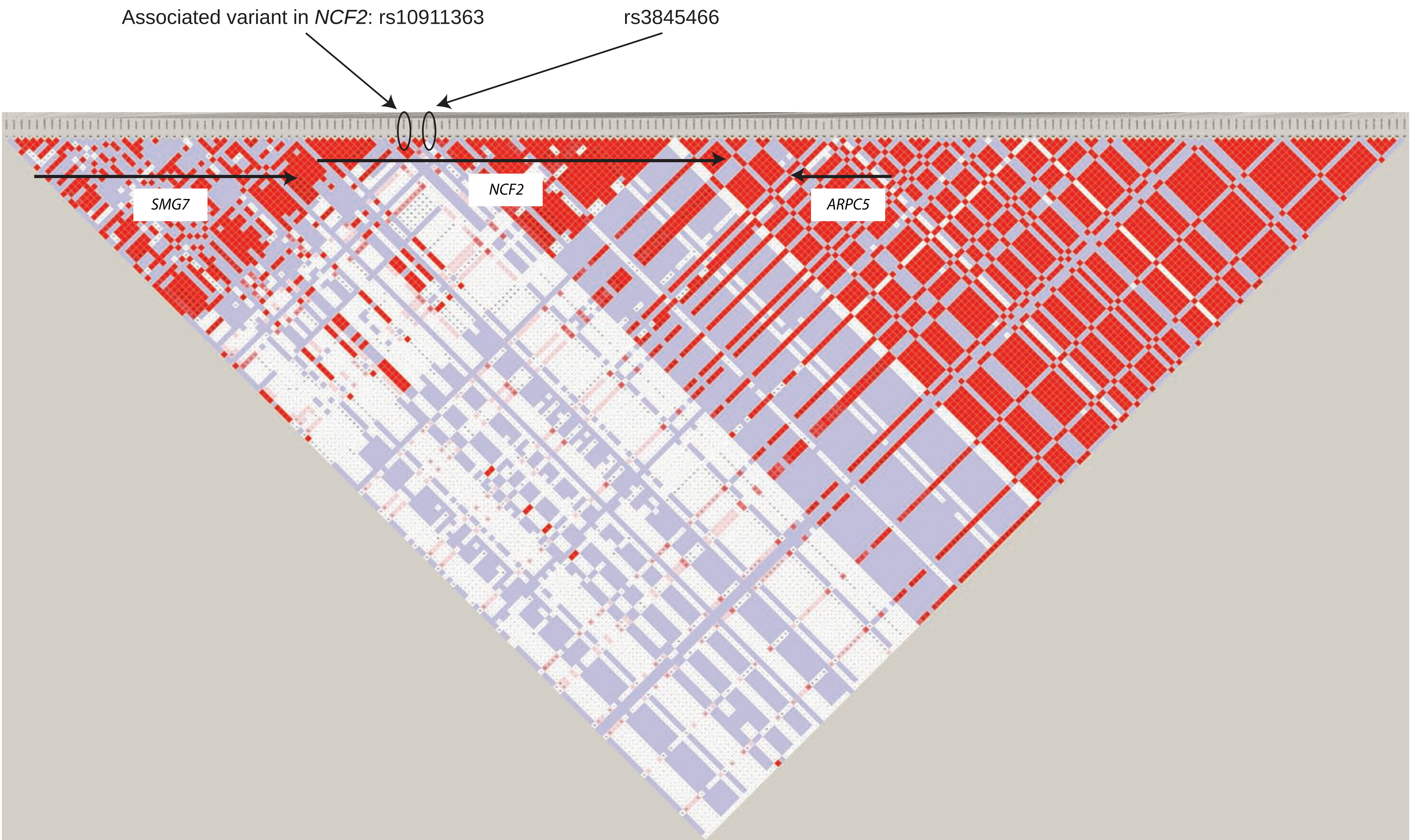

Figure S2A: LD pattern in 200 kb region around rs10911363

(Chr 1: 181,716,380-181,916,379- HAPMAP Data Rel 27 phase II+III Feb09, NCBI assembly dbSNP 126)

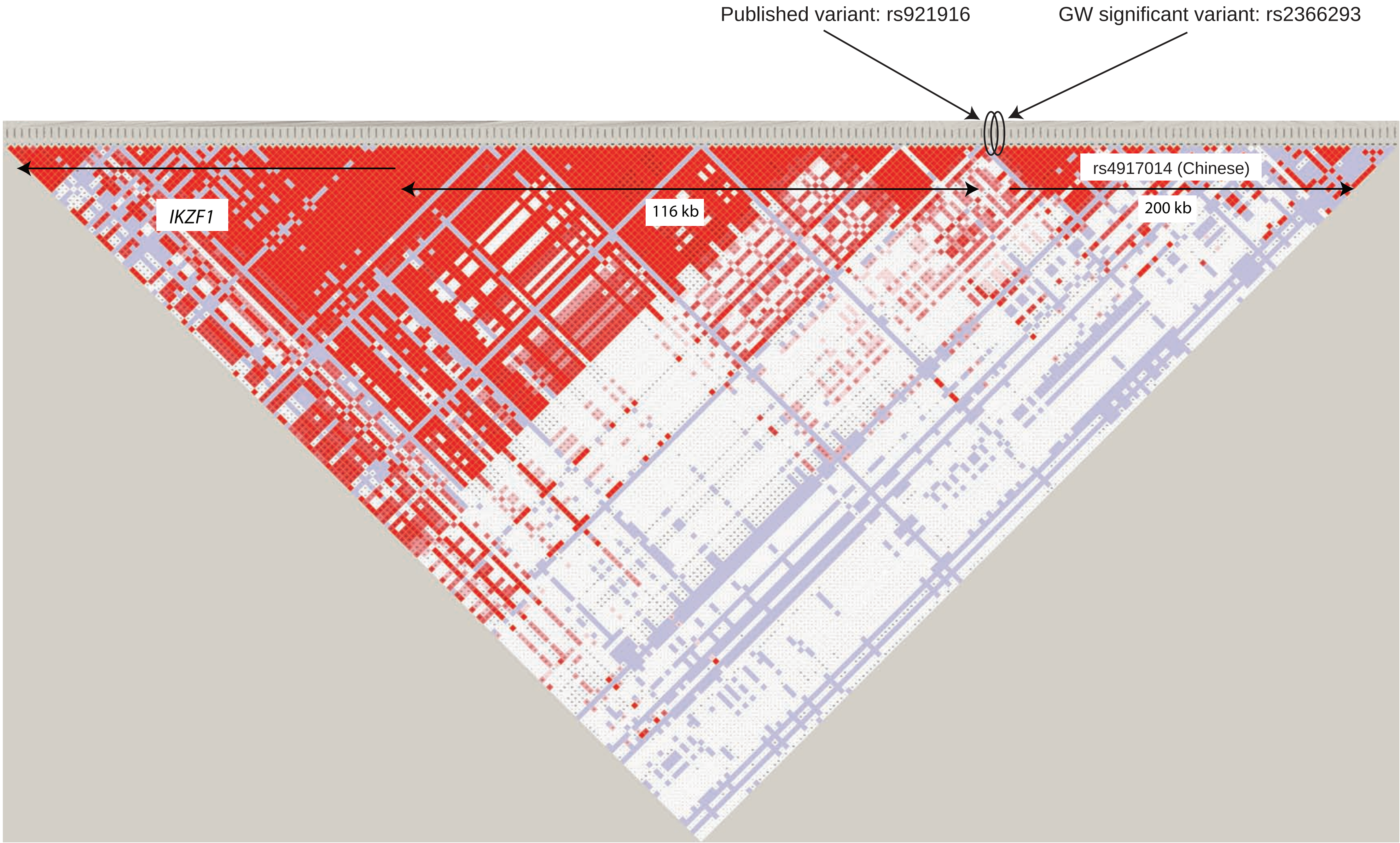

Figure S2B: LD pattern in 155.8 kb region around rs2366293

(Chr 7: 50,120,474-50,276,274 - HAPMAP Data Rel 27 phase II+III Feb09, NCBI assembly dbSNP 126)

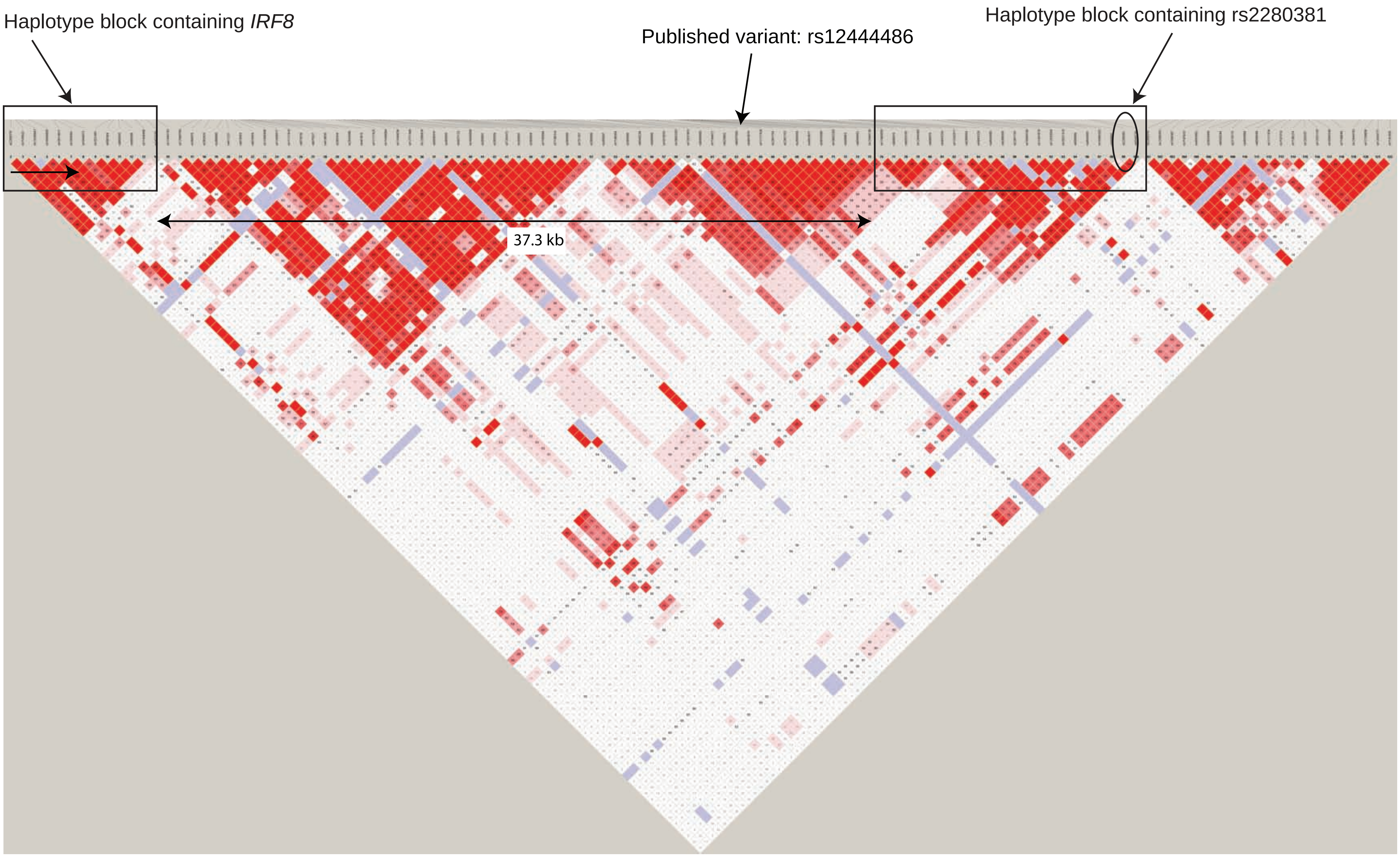

Figure S2C: LD pattern in 100 kb region around the gap between *IRF8* and rs2280381
(Chr 16: 84500150-845600149 - HAPMAP Data Rel 27 phase II+III Feb09, NCBI assembly dbSNP 126)

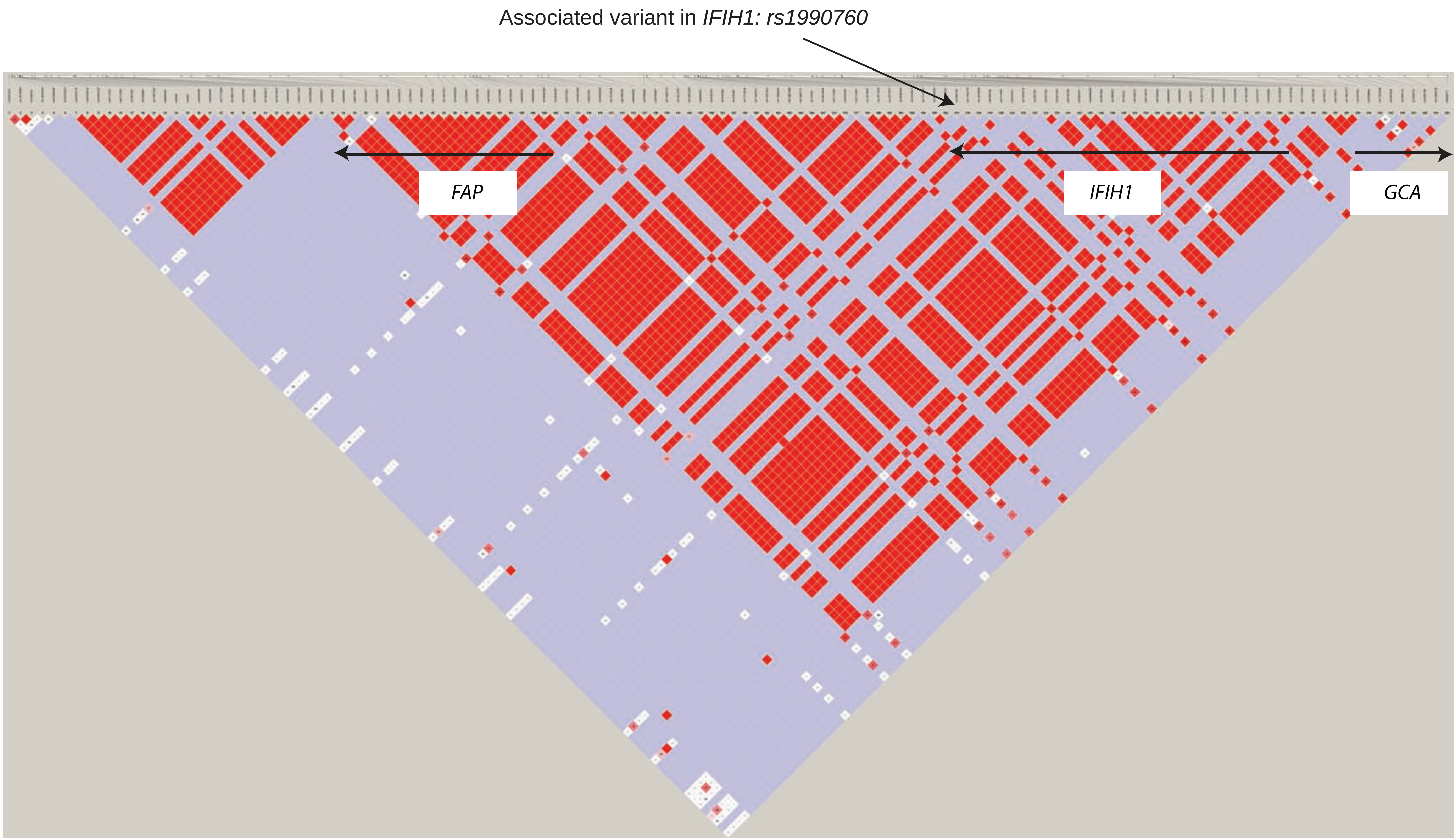

Figure S2D: LD pattern in 250kb region around rs1990760

(Chr 2: 162,700,000-162,949,999 - HAPMAP Data Rel 27 phase II+III Feb09, NCBI assembly dbSNP 126)

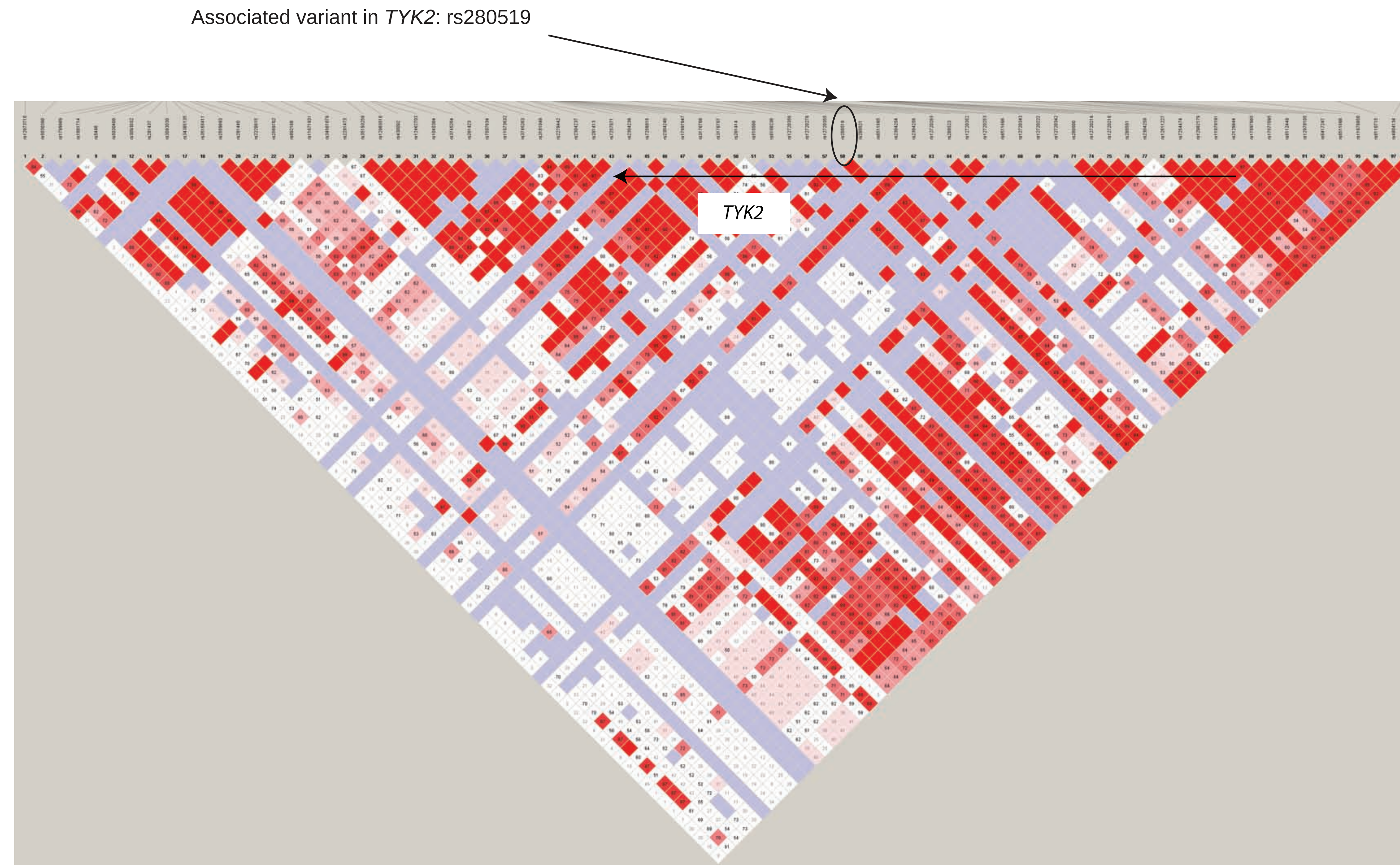

Figure S2E: LD pattern in 200 kb region around rs280519

(Chr 19: 10,233,933-10433933 - HAPMAP Data Rel 27 phase II+III Feb09, NCBI assembly dbSNP 126)
