# Supplementary material for: Association of NCF2, IKZF1, IRF8, IFIH1, and TYK2 with Systemic Lupus Erythematosus
Source: PLoS Genet. 2011 Oct 27;7(10):e1002341. doi: 10.1371/journal.pgen.1002341 (PMC3203198; doi:10.1371/journal.pgen.1002341)
Supplement: Table S1 — Composition of the study cohorts used. Each of the seven groups of samples included in this manuscript was independent from each other. In the UK population, direct genotyping was carried out on UK casesa and samples from the British Birth Control Cohorta (B58BCC). Genotypes from the WTCCC2b were used as out-of-study controls. The published data used n the meta-analysis described in this current manuscript was derived from US and Swedish samples. The US cohortc consisted of samples included in a GWAS and additional non-GWAS'd samples used just for the replication study, as described by Gateva et al. (2009) [4]. Full details of the Swedish (SWE)b replication samples are also described in Gateva et al (2009) [4]. (DOC) [file pgen.1002341.s004.doc]

**Table S**1: Composition of the study cohorts used

| **Study** | **Origin of Samples** | **SLE cases** | | |  | **Control samples** | | | | |
| --- | --- | --- | --- | --- | --- | --- | --- | --- | --- | --- |
|  |  | **UK**^a^ | **US^c^** | **SWE**^d^ |  | **UK (B58BCC)**^a^ | **UK (WTCCC2)**^b^ | | **US**^c^ | **SWE**^d^ |
| **UK** | UK | 905 |  |  |  | 68 | | 5483 |  |  |
| **US GWAS** | Gateva *et al* (2009) |  | 1310 |  |  |  | |  | 7859 |  |
| **US replication** | Gateva *et al* (2009) |  | 1129 |  |  |  | |  | 2991 |  |
| **SWE replication** | Gateva *et al* (2009) |  |  | 834 |  |  | |  |  | 1338 |
| **Total** |  | 905 | 2439 | 834 |  | 5551 | | | 10850 | 1338 |
