# Supplementary material for: Association of NCF2, IKZF1, IRF8, IFIH1, and TYK2 with Systemic Lupus Erythematosus
Source: PLoS Genet. 2011 Oct 27;7(10):e1002341. doi: 10.1371/journal.pgen.1002341 (PMC3203198; doi:10.1371/journal.pgen.1002341)
Supplement: Table S2 — Quality control of genotype data and imputation boundaries for WTCCC2 control samples. The position of each variant (column “Pos”) is given using NCBI Build 36. The number of WTCCC2 control samples is given for each variant in the column marked “WTCCC2 samples.” (DOC) [file pgen.1002341.s005.doc]

***Table S2: Quality control of genotype data and imputation boundaries for WTCCC2 control samples***

| **Marker** | **Chrom** | **Pos** | **Alleles (MA/Maj)** | **MAF cases** | **MAF controls** | **WTCCC2**  **samples** | **Imputation**  **Boundaries** | **Locus** | **HWE cases** | **HWE controls** | **% GT UK cases**  **(n = 870)** | **Reason for**  **withdrawal from analysis** |
| --- | --- | --- | --- | --- | --- | --- | --- | --- | --- | --- | --- | --- |
| rs1003355 | 17 | 46100065 | C/C | n/a | n/a | n/a | 46050064-46150064 | *ABCC3* | 1 | 1 | 0.96 | Failed typing |
| rs10742326 | 11 | 34766586 | A/G | 0.40 | n/a | n/a | 34716585-34816585 | *APIP* | 0.569 | 1 | 0.95 | Bad qual imputation |
| rs10516487 | 4 | 102970099 | T/C | 0.29 | 0.32 | 5392 |  | *BANK1* | 0.869 | 0.4890 | 0.95 |  |
| rs4748857 | 10 | 23639564 | T/C | 0.23 | 0.08 | 1009* | 23589563-23689563 | *C10ORF67* | 0.435 | 1 | 0.95 | Bad qual imputation |
| rs17696736 | 12 | 110971201 | G/A | 0.47 | 0.43 | 5392 |  | *C12ORF30* | 0.891 | 0.267 | 0.95 |  |
| rs6438700 | 3 | 123355515 | T/C | 0.18 | 0.18 | 5392 |  | *CASR* | 1 | 0.406 | 0.95 |  |
| rs641153 | 6 | 32022159 | T/C | 0.07 | 0.08 | 5239* | 31972158-32072158 | *CFB* | 0.791 | 0.0245 | 0.94 |  |
| rs12708716 | 16 | 11087374 | G/A | 0.33 | 0.35 | 5390* | 11037373-11137373 | *CLEC16A* | 0.396 | 0.788 | 0.95 |  |
| rs1861525 | 7 | 25128127 | G/A | 0.05 | 0.05 | 5392 |  | *CYCS* | 1.437E-18 | 0.281 | 0.93 |  |
| rs10156091 | 7 | 8153619 | T/C | 0.11 | 0.11 | 5392 |  | *ICA1* | 0.488 | 1 | 0.95 |  |
| rs1990760 | 2 | 162832297 | C/T | 0.38 | 0.39 | 5392 |  | *IFIH1* | 0.652 | 1 | 0.94 |  |
| rs3024505 | 1 | 205006527 | T/C | 0.17 | 0.16 | 5392 |  | *IL10* | 0.553 | 0.0219 | 0.95 |  |
| rs3212227 | 5 | 158675528 | C/A | 0.18 | 0.20 | 5392 |  | *IL12B* | 0.232 | 0.439 | 0.94 |  |
| rs1874791 | 1 | 67579020 | T/C | 0.19 | 0.17 | 4579* | 67529020-67629020 | *IL12RB2* | 0.00577 | 9.74E-06 | 0.94 |  |
| rs2280381 | 16 | 84576134 | G/A | 0.35 | 0.38 | 5392 |  | *IRF8* | 0.097 | 0.751 | 0.94 |  |
| rs849142 | 7 | 28152416 | G/A | 0.48 | 0.52 | 5193* | 28102415-28202415 | *JAZF1* | 0.193 | 0.071 | 0.95 |  |
| rs9782955 | 1 | 234106500 | T/C | 0.24 | 0.25 | 5392 |  | *LYST* | 0.347 | 0.797 | 0.95 |  |
| rs10911363 | 1 | 181816380 | T/G | 0.31 | 0.27 | 5392 |  | *NCF2* | 0.0483 | 0.139 | 0.95 |  |
| rs2022013 | 1 | 181620476 | G/A | 0.40 | 0.42 | 5392 |  | *NMNAT2* | 0.618 | 0.123 | 0.95 |  |
| rs11951576 | 5 | 6784413 | T/C | 0.31 | 0.31 | 5392 |  | *POLS* | 0.873 | 0.657 | 0.95 |  |
| rs3184504 | 12 | 110368991 | T/C | 0.51 | 0.49 | 5392 |  | *SH2B3* | 0.946 | 0.445 | 0.95 |  |
| rs428073 | 12 | 117167134 | C/T | 0.29 | 0.32 | 5392 |  | *TAOK3* | 0.213 | 0.729 | 0.94 |  |
| rs6889239 | 5 | 150437964 | T/C | 0.29 | 0.23 | 5228* | 150387963-150487963 | *TNIP1* | 0.559 | 0.0747 | 0.94 |  |
| rs280519 | 19 | 10333933 | G/A | 0.49 | 0.53 | 5392 |  | *TYK2* | 0.410 | 0.113 | 0.94 |  |
| rs11755393 | 6 | 34932614 | G/A | 0.43 | 0.34 | 5392 |  | *UHRF1BP1* | 2.30E-06 | 0.468 | 0.96 | HWE |
| rs497273 | 12 | 119689065 | G/C | 0.35 | 0.36 | 5178* | 119639064-119739064 | *UNQ1887* | 0.499 | 0.0956 | 0.94 |  |
| rs2366293 | 7 | 50198374 | G/C | 0.16 | 0.13 | 4851* | 50148373-50248373 | *IKZF1* | 0.382 | 0.0114 | 0.94 |  |
