# Supplementary material for: Association of NCF2, IKZF1, IRF8, IFIH1, and TYK2 with Systemic Lupus Erythematosus
Source: PLoS Genet. 2011 Oct 27;7(10):e1002341. doi: 10.1371/journal.pgen.1002341 (PMC3203198; doi:10.1371/journal.pgen.1002341)
Supplement: Table S3 — Power calculations. aNovel associations in this study (5×10^−8^). The OR, as a measure of effect size was taken from the case-control association study. The power was calculated according to Purcell et al 2003 (http://bioinformatics.oxfordjournals.org/content/19/1/149.full.pdfhtml), using a disease prevalence of 0.0002. The risk allele frequency was calculated in both cases and controls. GRR (AB) = (ABcase/AAcase)/(ABcontrol/AAcontrol) and GRR (AA) = (BBcase/AAcase)/(BBcontrol/AAcontrol). (DOC) [file pgen.1002341.s006.doc]

**Table S**3: Power Calculations

| **Marker** |  | **Power** | **Risk allele** | **OR** | **Risk**  **allele Freq** | **GRR (AB)** | **GRR (BB)** | **GT counts case (n=870)** | | |  | **GT counts controls (n=5551)** | | |
| --- | --- | --- | --- | --- | --- | --- | --- | --- | --- | --- | --- | --- | --- | --- |
|  |  |  |  |  |  |  |  | **BB** | **AB** | **AA** |  | **BB** | **AB** | **AA** |
| rs10911363^a^ | *NCF2* | >95% | T | 1.23 | 0.28 | 1.12 | 1.63 | 99 | 339 | 417 |  | 419 | 2090 | 2874 |
| rs2366293^a^ | *IKZF1* | <93% | G | 1.27 | 0.14 | 1.38 | 1.09 | 19 | 242 | 589 |  | 108 | 1087 | 3648 |
| rs2280381^a^ | *IRF8* | >54% | A | 1.12 | 0.62 | 0.96 | 1.17 | 366 | 369 | 115 |  | 2097 | 2593 | 772 |
| rs1990760^a^ | *IFIH1* | >54% | T | 1.09 | 0.61 | 1.10 | 1.24 | 343 | 388 | 114 |  | 2025 | 2589 | 835 |
| rs280519^a^ | *TYK2* | >92% | A | 1.20 | 0.48 | 1.31 | 1.43 | 221 | 434 | 192 |  | 1247 | 2666 | 1548 |
| rs6889239* | *TNIP1* | >99% | C | 1.35 | 0.24 | 1.32 | 1.60 | 69 | 341 | 441 |  | 305 | 1823 | 3114 |
| rs849142 | *JAZF1* | >62% | A | 1.16 | 0.49 | 1.32 | 1.28 | 214 | 449 | 190 |  | 1247 | 2526 | 1412 |
| rs3024505 | *IL10* | >21% | T | 1.07 | 0.17 | 1.08 | 1.18 | 23 | 256 | 585 |  | 126 | 1534 | 3788 |
| rs428073* | *TAOK3* | >48% | T | 1.12 | 0.69 | 0.96 | 1.13 | 432 | 339 | 80 |  | 2549 | 2370 | 538 |
| rs17696736 | *C12ORF30* | >97% | G | 1.18 | 0.44 | 1.21 | 1.49 | 197 | 433 | 230 |  | 1003 | 2718 | 1742 |
| rs9782955 | *LYST* | >12% | C | 1.06 | 0.75 | 0.91 | 1.00 | 504 | 303 | 53 |  | 3096 | 2038 | 326 |
| rs187479* | *IL12RB2* | low GRR AB | T | 1.19 | 0.18 | 0.99 | 1.23 | 38 | 242 | 575 |  | 188 | 1495 | 3509 |
| rs497273 | *UNQ1887* | <10% | G | 1.03 | 0.64 | 1.03 | 1.03 | 360 | 384 | 110 |  | 2168 | 2319 | 684 |
| rs1861525 | *CYCS* | low risk AF | G | 1.03 | 0.05 | 0.56 | 8.47 | 20 | 42 | 783 |  | 15 | 476 | 4975 |
| rs11951576 | *POLS* | <10% | C | 1.02 | 0.69 | 0.96 | 1.00 | 410 | 363 | 83 |  | 2581 | 2365 | 520 |
| rs641153 | *CFB* | 52% | C | 1.23 | 0.92 | 2.32 | 2.76 | 743 | 111 | 3 |  | 4401 | 781 | 49 |
| rs6438700 | *CASR* | too low AF | C | 1.01 | 0.82 | 1.08 | 1.07 | 579 | 251 | 27 |  | 3692 | 1589 | 185 |
| rs3212227 | *IL12B* | >55% | A | 1.15 | 0.81 | 0.88 | 1.08 | 584 | 236 | 31 |  | 3464 | 1721 | 198 |
| rs3184504 | *SH2B3* | >66% | T | 1.09 | 0.49 | 1.15 | 1.28 | 227 | 434 | 193 |  | 1291 | 2752 | 1403 |
| rs12708716 | *CLEC16A* | >38% | A | 1.10 | 0.65 | 0.97 | 1.13 | 391 | 364 | 100 |  | 2299 | 2496 | 666 |
| rs10516487 | *BANK1* | 59% | C | 1.13 | 0.69 | 1.23 | 1.35 | 431 | 358 | 69 |  | 2573 | 2336 | 556 |
| rs10156091 | *ICA1* | low risk AF | T | 1.03 | 0.11 | 1.06 | 0.80 | 8 | 172 | 679 |  | 64 | 1038 | 4361 |
