# Supplementary material for: Association of NCF2, IKZF1, IRF8, IFIH1, and TYK2 with Systemic Lupus Erythematosus
Source: PLoS Genet. 2011 Oct 27;7(10):e1002341. doi: 10.1371/journal.pgen.1002341 (PMC3203198; doi:10.1371/journal.pgen.1002341)
Supplement: Table S4 — Results of weighted meta-analysis using METAL and calculation of combined OR. The total number of individuals included in the meta-analysis was: 870 UK SLE cases and 5,551 UK control samples and 3,273 SLE cases and 12,188 controls taken from the US/SWE out-of-study cohort [1]. The risk allele frequency quoted is that from the UK cases. The column marked Het P-value represents the test for heterogeneity of odds ratios between the UK and published dataset and the column marked ORcomb represents the OR in the combined dataset, calculated using METAL. The column marked Direction of Effect demonstrates that the effect for each quoted allele is the same for the UK and US/SWE datasets. (DOC) [file pgen.1002341.s007.doc]

**Table S4: Results of weighted meta-analysis using METAL and calculation of combined OR**

| **SNP** | **Gene** | **Risk Allele** | **Risk Allele Freq** | **Meta-Analysis using METAL** | | | |
| --- | --- | --- | --- | --- | --- | --- | --- |
|  |  |  |  | **OR_comb_** | ***P* value** | **Het *P-*value** | **Direction of Effect** |
| rs10911363 | *NCF2* | T | 0.31 | 1.20 | 1.15x10^-10^ | 0.873 | ++ |
| rs2366293 | *IKZF1* | G | 0.16 | 1.21 | 5.77x10^-9^ | 0.846 | ++ |
| rs2280381 | *IRF8* | A | 0.65 | 1.16 | 4.57x10^-8^ | 0.259 | ++ |
| rs1990760 | *IFIH1* | T | 0.63 | 1.15 | 5.00x10^-8^ | 0.273 | ++ |
| rs280519 | *TYK2* | A | 0.52 | 1.17 | 1.56x10^-7^ | 0.436 | ++ |
| rs6889239 | *TNIP1* | C | 0.29 | 1.27 | 7.61x10^-17^ | 0.931 | ++ |
| rs849142 | *JAZF1* | A | 0.52 | 1.17 | 3.64x10^-10^ | 0.171 | ++ |
| rs3024505 | *IL10* | T | 0.17 | 1.20 | 9.20x10^-8^ | 0.0564 | ++ |
| rs428073 | *TAOK3* | T | 0.71 | 1.15 | 3.86x10^-7^ | 0.214 | ++ |
| rs17696736 | *C12ORF30* | G | 0.47 | 1.14 | 4.43x10^-7^ | 0.225 | ++ |
| rs9782955 | *LYST* | C | 0.76 | 1.15 | 3.05x10^-6^ | 0.0369 | ++ |
| rs1874791 | *IL12RB2* | T | 0.19 | 1.17 | 3.29x10^-6^ | 0.0195 | ++ |
| rs497273 | *UNQ1887* | G | 0.65 | 1.12 | 9.02x10^-6^ | 0.0195 | ++ |
| rs1861525 | *CYCS* | G | 0.05 | 1.31 | 2.24x10^-5^ | 0.0379 | ++ |
| rs11951576 | *POLS* | C | 0.69 | 1.11 | 8.09x10^-5^ | 1 | ++ |
| rs641153 | *CFB* | C | 0.93 | 1.27 | 6.98x10^-6^ | 0.771 | ++ |
| rs6438700 | *CASR* | C | 0.82 | 1.14 | 9.68x10^-5^ | 0.0165 | ++ |
| rs3212227 | *IL12B* | A | 0.83 | 1.13 | 8.84x10^-5^ | 0.784 | ++ |
| rs3184504 | *SH2B3* | T | 0.51 | 1.11 | 2.35x10^-5^ | 0.864 | ++ |
| rs12708716 | *CLEC16A* | A | 0.67 | 1.12 | 1.71x10^-5^ | 0.514 | ++ |
| rs10516487 | *BANK1* | C | 0.71 | 1.12 | 5.97x10^-5^ | 0.877 | ++ |
| rs10156091 | *ICA1* | T | 0.11 | 1.15 | 7.16x10^-3^ | 0.160 | ++ |
| rs2022013 | *NMNAT2* | A | 0.60 | 1.09 | 7.43x10^-3^ | 0.375 | ++ |
