# Supplementary material for: Association of NCF2, IKZF1, IRF8, IFIH1, and TYK2 with Systemic Lupus Erythematosus
Source: PLoS Genet. 2011 Oct 27;7(10):e1002341. doi: 10.1371/journal.pgen.1002341 (PMC3203198; doi:10.1371/journal.pgen.1002341)
Supplement: Table S5 — Association Analysis in UK, and US-Swedish Populations for Markers Previously Showing Genome-Wide Significance (P<5×10^−8^). a For sample numbers see reference [1] and Table S1 (US GWAS: 1,310 cases and 7,859 controls; US replication cohort: 1,129 cases and 2,991 controls; Swedish replication cohort: 834 cases and 1,388 controls). b The risk allele frequency was calculated in control individuals. c Unpublished data. (DOC) [file pgen.1002341.s008.doc]

**Table S5: Association Analysis in UK, and US-Swedish Populations for Markers Previously Showing Genome-Wide Significance (*P*<5 x 10^-8^**)

| **MARKER** | **Locus** | **Risk allele** | **UK population**  ***870 cases, 5551 controls*** | | | | |  | ***P* value (US/SWE)^a^**  ***3273 cases, 12188 controls*** | | **Combined Analysis**  **Fisher’s test**  ***P* value** |
| --- | --- | --- | --- | --- | --- | --- | --- | --- | --- | --- | --- |
|  |  |  | **Freq risk allele^b^** | | **OR** | ***P* value** | |  | **OR** | ***P* value** |  |
| rs6889239 | *TNIP1* | *C* | *0.23* | 1.30 | | | 9.06x10^-6^ |  | 1.26 | 1.45x10^-12^**^c^** | 1.31x10^-17^ |
| rs849142 | *JAZF1* | *A* | *0.48* | 1.13 | | | 0.0243 |  | 1.19 | 1.50x10^-9^ | 3.65x10^-11^ |
| rs3024505 | *IL10* | *T* | *0.16* | 1.09 | | | 0.209 |  | 1.19 | 4.00x10^-8^ | 8.36x10^-9^ |
