# Supplementary material for: Association of NCF2, IKZF1, IRF8, IFIH1, and TYK2 with Systemic Lupus Erythematosus
Source: PLoS Genet. 2011 Oct 27;7(10):e1002341. doi: 10.1371/journal.pgen.1002341 (PMC3203198; doi:10.1371/journal.pgen.1002341)
Supplement: Text S1 — This file contains supplementary genomic and functional details about the five SLE susceptibility genes reaching genome-wide levels of significance. (DOC) [file pgen.1002341.s009.doc]

**Text S1**

***NCF2*  (Neutrophil cytosolic factor 2)**

We independently replicated the association of the intron 2 SNP, rs19011363, in our UK cohort (*P*=3.02x10^-4^) and found a novel genome-wide significance by meta-analysis (*P*=2.87x10^-11^) (**Table 1**). As can be seen in **Figure S2A**, the coding region of *NCF2* is split between two large LD blocks. The associated variant, rs19011363, sits within a region of low LD, flanked by these two LD blocks.

***IKZF1*** (**Ikaros family zinc finger 1)**

*IKZF1* is part of the five-member Ikaros family of transcription factors. In our UK cohort we discovered a novel association for rs2366393 located 116kb upstream of *IKZF1* (*P*=8.77x10^-4^). Following meta-analysis, the association surpassed genome-wide significance (*P*=1.10x10^-10^) (**Table 1**). Therefore there are two SNPs in the upstream region of *IKZF1* which show association with SLE in Europeans: rs2366293 from this current manuscript (P_combined_=1.10x10^-10^) and rs921916 from the published US/SWE study (*P*_combined_=2.0x10^-6^) (**Figure S2C**)**[1]**. There was a third SNP, rs4917014, that showed association with SLE in a Chinese GWAS (P_GWAS_=2.93x10^-06^)**[5]**. This Chinese variant rs4917014 is more distal than rs2366293/rs921916, being located ~200 kb upstream of *IKZF1*. However, we propose that rs4917014 represents a separate signal from that seen from the region around rs2366293, because there is a breakdown in LD between the two regions in both the European or Chinese (CHB) HAPMAP samples (r^2^<0.002). Furthermore, there are population-differences in minor allele frequencies for the three SNPs. The minor allele frequency for the Chinese risk variant, rs4917014, is consistent across the CEU, YRI and CHB/JPT (ASN) populations, whereas for both European risk variants (rs2366293 and rs921916), there is an ~8-fold difference in the minor allele between the CEPH and CHB HAPMAP populations. Finally, there was no reported allele-specific association for rs4917014 with levels of *IKZF1***[6]**, nor is there a consistent trend in increased expression of IKZF1 in the CEU, YRI CHB/JPT (ASN) HAPMAP populations using publically available data (data not shown).

***IFR8*  (Interferon regulatory factor 8)**

We identified a variant, rs2280381, showing association in our UK cohort (*P*=0.0368) and genome-wide significance by meta-analysis (*P*=1.24x10^-08^) (**Table 1**). rs2280381 is 64kb downstream of *IRF8*, with evidence of long-range LD (D’>0.5) between the LD blocks containing rs2280381 and that containing the coding region of *IRF8* (**Figure S2C**)**[1]***.* The associated variant from this manuscript, rs2280381, is located 26.5 kb away from a published association using genotypes for rs12444486 in samples used for an SLE GWAS (P_combined_=1.9x10^-07^) (**Figure S2C**)**[1]**. Both the directly-typed SNP from this publication and the imputed rs12444486 genotypes from the published data are located in the downstream flanking region of *IRF8.* Since both SNPs are in strong LD (D’=0.463), this increases the evidence for the genetic contribution to SLE susceptibility in the 3’ flanking region of *IRF8.*

In addition to reporting an allele-specific increase in gene expression for the risk allele of rs2280381 we used data taken from chromatin analysis methods, including Chip-Seq, from the UCSC ENCODE regulation supertrack, to show that region around rs2280381 has a potentially active role in transcription. This is because the 25 bp region spanning rs2280381 contains histone marks for H3K4Me1 (peak P(-log10)=30.6), H3K27Ac (peak P(-log10)=44.75) and H3K4Me3 (peak P(-log10)=12.3)**[7]**. When taken together these two sets of results reinforce the suggestion that the region around rs2280381 is not only transcriptionally “open” but is also correlated with increased expression of the IFN pathway regulator, *IRF8*.

***IFIH1* (interferon-induced helicase C domain-containing protein 1)**

We found an association at *IFIH1* in our UK cohort (*P*=0.0487), which crossed the threshold for genome-wide significance in the UK/US/SWE dataset (*P*=1.63x10^-8^). However, the pattern of LD in *IFIH1* for SLE is complex (**Figure S2D**), since the conserved allele of three rare variants in *IFIH1* showed allele-specific increases in *IFIH1* expression, despite not being in LD with the common allele of rs1990760**[3]**. In T1D, logistic regression analysis in a case-control cohort demonstrated that rs1990760 was the common variant best explaining the common association across an LD block covering *IFIH1* and three other genes (**Figure S2D**)**[4]**.

The associated variant, rs1990760, is an Ala946Thr missense mutation found in exon 14, which exchanges a polar amino acid side chain for a hydrophic chain, but is tolerated by the protein (PSIC score difference=0.393) (SIFT, Polyphen). The conservation of the 946Ala residue across vertebrates**[4]**, suggests a functional role for the protein.

***TYK2*  (Tyrosine kinase 2)**

In our UK cohort we found a significant association for an intronic variant, rs280519 (*P*=5.24x10^-4^), which crossed the threshold for GW significance after meta-analysis (*P*=3.88x10^-8^) (**Table 1**). The location of the associated variant, rs280519 within *TYK2* is shown in **Figure S2E**.

**Reference List**

1. Gateva, V., et al., A large-scale replication study identifies TNIP1, PRDM1, JAZF1, UHRF1BP1 and IL10 as risk loci for systemic lupus erythematosus. Nat.Genet., 2009. 41(11): p. 1228-1233.

2. Nayak, R.R., et al., Coexpression network based on natural variation in human gene expression reveals gene interactions and functions. Genome Res, 2009. 19(11): p. 1953-62.

3. Downes, K., et al., Reduced expression of IFIH1 is protective for type 1 diabetes. PLoS One, 2010. 5(9).

4. Smyth, D.J., et al., A genome-wide association study of nonsynonymous SNPs identifies a type 1 diabetes locus in the interferon-induced helicase (IFIH1) region. Nat Genet, 2006. 38(6): p. 617-9.

5. Han, J.W., et al., Genome-wide association study in a Chinese Han population identifies nine new susceptibility loci for systemic lupus erythematosus. Nat Genet, 2009. 41(11): p. 1234-7.

6. Hu, W., et al., Down-regulated expression of IKZF1 mRNA in peripheral blood mononuclear cells from patients with systemic lupus erythematosus. Rheumatol Int, 2010.

7. Birney, E., et al., Identification and analysis of functional elements in 1% of the human genome by the ENCODE pilot project. Nature, 2007. 447(7146): p. 799-816.
